# Clinical cure induced by pegylated interferon α-2b in the advantaged population of chronic hepatitis B virus infection: a retrospective cohort study

**DOI:** 10.3389/fcimb.2023.1332232

**Published:** 2024-01-16

**Authors:** Chaojing Wen, Yixuan Wang, Haoyue Tian, Yu Lei, Zhiyi Wang, Dachuan Cai, Zhi Zhou, Xiaofeng Shi

**Affiliations:** Department of Infectious Diseases, Key Laboratory of Molecular Biology for Infectious Diseases (Ministry of Education), Institute for Viral Hepatitis, The Second Affiliated Hospital of Chongqing Medical University, Chongqing, China

**Keywords:** clinical cure, chronic inactive hepatitis B virus carriers, nucleoside analog-experienced patients, HBsAg clearance, HBsAg seroconversion, pegylated interferon α-2b

## Abstract

**Background:**

Among the advantaged population with clinical cure of chronic hepatitis B, chronic inactive hepatitis B virus carriers (IHCs) and nucleoside analog-experienced patients have similar serological manifestations. This study established non-interferon-treated groups as controls to compare the efficacy of pegylated interferon α-2b (Peg-IFNα-2b) in achieving clinical cure between IHCs and nucleoside analog (NA)-experienced patients.

**Method:**

A total of 270 patients were enrolled in this observational study. The IHC cohort comprised 55 patients who received Peg-IFNα-2b (Peg-IFN group), and the other 70 patients did not receive any antiviral treatment (untreated group). Patients treated with NAs were divided into two groups: one group (70 patients) receiving NA add-on Peg-IFNα-2b therapy regimen (NA add-on Peg-IFN group) and another group (75 patients) receiving continuous NA monotherapy (NA group). The primary endpoints were hepatitis B surface antigen (HBsAg) clearance and HBsAg seroconversion at 48 weeks and 72 weeks.

**Results:**

At 48 weeks, 65.5% (36/55) and 52.9% (37/70) patients achieved HBsAg clearance in the Peg-IFN group and NA add-on Peg-IFN group, respectively (p = 0.156). HBsAg seroconversion was achieved in 47.3% (26/55) of the Peg-IFN group and 34.3% (24/70) of the NA add-on Peg-IFN group (p = 0.141). At the follow-up of 72 weeks, 36 patients in the Peg-IFN group achieved HBsAg loss (65.5%, 36/55), and 33 patients in the NA add-on Peg-IFN group achieved HBsAg clearance (47.1%, 33/70), which were significantly higher than in the Peg-IFN group (p = 0.041). The HBsAg seroconversion rates in the Peg-IFN group and NA add-on Peg-IFN group at 72 weeks were 45.5% (25/55) and 32.9% (23/70), respectively (p = 0.151). No patient achieved HBsAg clearance or seroconversion in the NA group and untreated group. Furthermore, the receiver operating characteristic curve showed baseline HBsAg< 72 IU/mL, and the decline of HBsAg of more than 80% and 98% from baseline to 12 and 24 weeks provided good predictions for HBsAg clearance. Meanwhile, 77% of patients with baseline HBsAg< 100 IU/mL achieved a clinical cure at 48 weeks.

**Conclusion:**

Peg-IFNα-2b results in a high rate of HBsAg clearance and seroconversion in both IHCs and NA-experienced patients, especially for those patients who have HBsAg below 100 IU/mL.

## 1 Introduction

Chronic hepatitis B (CHB) is one of the leading causes of hepatocellular carcinoma (HCC), liver cirrhosis, and liver-related death worldwide (Papatheodoridis et al., 2015). Therefore, the goal of antiviral treatment for hepatitis B patients is to delay or even prevent disease progression by maximally inhibiting HBV DNA replication and improving biochemical indicators (Viganò et al., 2018). A large cohort study (Yip et al., 2019) confirmed that the risk of HCC development was lower in patients who cleared hepatitis B surface antigen (HBsAg). Therefore, global experts have reached a consensus that HBsAg loss with or without seroconversion to hepatitis B surface antibody (HBsAb) is an ideal endpoint of antiviral therapy for CHB patients (Sarin et al., 2016; Lampertico et al., 2017; Terrault et al., 2018).

Currently, the antiviral drugs widely used in clinical are still nucleoside analogs (NAs). NAs have the characteristics of high antiviral potency and a high genetic barrier to drug-resistant mutations, so they are more suitable for chronic hepatitis B patients to take for a long time. With their long-term antiviral treatment, CHB patients gradually evolved into an advantaged group with low HBV DNA load, negative hepatitis B e-antigen (HBeAg), and HBsAg of less than 1,500 IU/mL (Hu et al., 2018). This advantaged group can obtain a clinical cure by Peg-IFN combination in order to reach the ideal treatment endpoint, and the rate of HBsAg disappearance can reach above 30% after 48-week treatment (Boglione et al., 2016; Wu et al., 2020).

In those with chronic hepatitis B virus infection, there are some groups of patients who do not meet the antiviral treatment criterion according to the relevant international guidelines (Sarin et al., 2016; Lampertico et al., 2017; Terrault et al., 2018). Among them are chronic inactive hepatitis B virus carriers (IHCs), which are typically characterized by negative HBeAg, low or undetectable HBV DNA, continuous normal alanine aminotransferase (ALT) or aspartate aminotransferase (AST) levels, and rare pathological changes in liver tissues. However, this status is not stable. A prospective follow-up study (Chu and Liaw, 2007) showed that the cumulative probability of hepatitis reactivation in IHCs increases annually, reaching approximately 20.2% over 25 years. Additionally, IHCs exhibit a higher risk of HCC, cirrhosis, and liver disease-related death than patients without HBV infection (Chen et al., 2010). The management of this population has become a hot spot in clinical research. Importantly, some studies (Chang et al., 2010; Marcellin et al., 2013) exhibited that selecting appropriate IHCs to receive Peg-IFN therapy can also lead to a high clinical cure.

Currently, those studied are conducted independently and lack Peg-IFN-free control. Hence, the primary purpose of this study was to compare the efficacy of pegylated interferon α-2b (Peg-IFNα-2b) in achieving a functional cure between the IHCs and NA-experienced patients in the real world by setting up Peg-IFN-free patients as controls.

## 2 Method

### 2.1 Patient selection

This observational cohort study was based on real-world data to compare the efficacy of Peg-IFNα-2b in achieving clinical cure between IHCs and NA-experienced patients. The criteria were as follows: the IHCs as patients who were HBsAg positive for more than 6 months, had HBsAg level<1,000 IU/mL, were HBeAg-negative/anti-HBe-positive, had low HBV-DNA levels (<2,000 IU/mL), had normal ALT levels, and did not receive any antiviral treatment according to the Prevention and Treatment Guidelines for Chronic Hepatitis B (2019 edition). The NA-experienced patients have been taking nucleoside analogs for more than 1 year and meet the following criteria: HBsAg level<1,000 IU/mL, HBeAg-negative (including HBeAg-negative patients or HBeAg-positive patients who became HBeAg-negative after NA antiviral treatment), and HBV-DNA levels below 2,000 IU/mL. Exclusion criteria were as follows: 1) co-infected with hepatitis A, C, D, or human immunodeficiency virus; 2) decompensated liver diseases, alcohol or drug abuse, autoimmune diseases, severe metabolic diseases, HCC, or tumors in any systems; 3) severe complications in any organ; and 4) pregnant or lactating women.

This real-world observational research was conducted at the Second Affiliated Hospital of Chong Qing Medical University in China from June 2019 to September 2021. The study complies with good clinical practice and the Declaration of Helsinki and was approved by the Ethics Committee of the Second Affiliated Hospital of Chong Qing Medical University.

### 2.2 Study method

**Figure 1:**
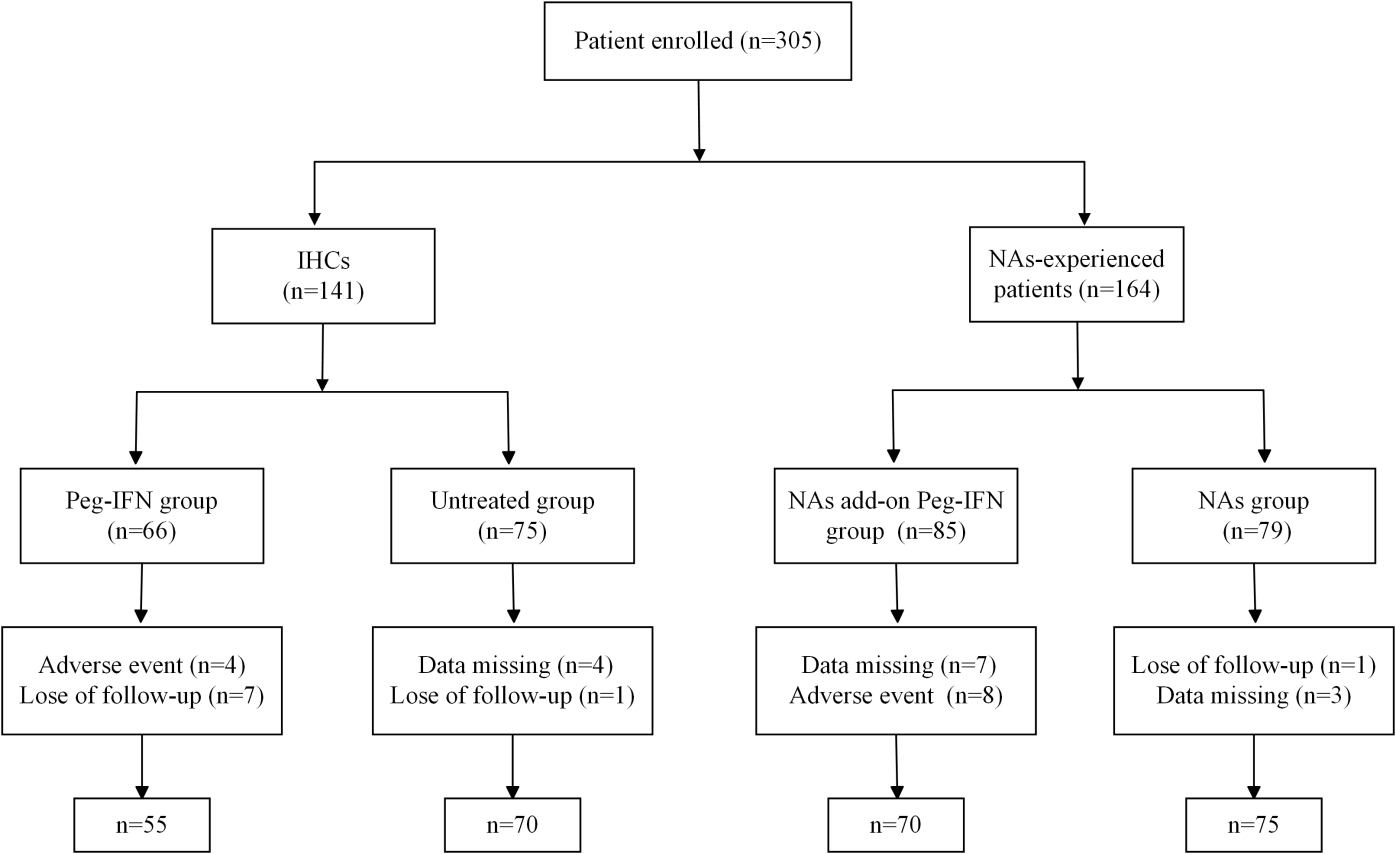
Flow diagram of patient enrollment in this study. IHCs, inactive hepatitis B surface antigen (HBsAg) carriers; NAs, nucleoside analogs.

According to the inclusion criteria, the IHC cohort was divided into the Peg-IFN group and the untreated group. Patients treated with nucleoside analogs were also divided into two groups: one group of patients receiving either Peg-IFNα-2b add-on therapy once weekly or an ongoing NA regimen (NA add-on Peg-IFN group) and another group receiving continuous NA monotherapy (NA group) (**Figure 1**). Peg-IFNα-2b was injected subcutaneously 180 μg once a week, and the treatment endpoint was Peg-IFNα-2b treatment for 48 weeks or obtained HBsAg clearance. The primary endpoints were HBsAg clearance, HBsAg seroconversion, and virological response at 48 weeks and 72 weeks. The second endpoints were biochemical indicators (ALT and AST), blood routine, blood sugar, and thyroid function, which can reflect the adverse effects of Peg-IFN.

### 2.3 Study assessment

Study assessments were based on the laboratory results and clinical and safety evaluations. Before initiating Peg-IFN treatment, it is essential to test antinuclear antibody, thyroid function, abdominal ultrasound, venous blood sugar, serum HBsAg level, HBV DNA, blood routine, alpha-fetoprotein (AFP), fibrosis score, and liver function. Additionally, serum HBsAg levels, anti-HBs levels, HBV DNA levels, neutrophil and platelet counts, and liver and kidney function were evaluated at 12 weeks, 24 weeks, 36 weeks, and 48 weeks. Quantitative detection of hepatitis B was performed using Abbott chemiluminescence automatic immunoassays (Architect i2000 HBsAg quantitative assays; Abbott Laboratories, Abbott Park, IL, USA). The detection limit for HBsAg was 0.05 IU/mL. Serum HBV DNA was detected using the TaqMan-based real-time polymerase chain reaction assay (Shanghai ZJ BioTech, Shanghai, China) with a limit of detection of 100 IU/mL. The samples with HBsAb levels greater than 10 IU/mL were considered positive. HBsAg loss was defined as an HBsAg concentration of less than 0.05 IU/mL. Serum ALT was assayed using an automatic biochemical analyzer (Roche, Basel, Switzerland) and presented as multiples of the upper limit of normal value (ULN) (men, 50 IU/L; women, 40 IU/L). Safety assessment included pyrexia, thrombocytopenia, neutropenia, leukopenia, alopecia, and headache.

### 2.4 Statistical analysis

The analyses were performed using the Statistical Package for Social Sciences (SPSS, version 25.0, Chicago, IL, USA). Based on the results of the Kolmogorov–Smirnov normality test, normal distribution variables are expressed as means ± standard deviations, and non-normal distribution continuous variables are expressed as median and interquartile ranges (Q1–Q3). Categorical variables are reported as counts and percentages. Differences from the baseline characteristics and treatment data were compared using a χ^2^ test for categorical variables or the one-way ANOVA for continuous variables. Additionally, univariable and multivariable analyses were adopted to analyze the predictors of HBsAg clearance. For continuous predictors, the receiver operating characteristic (ROC) curve analyses were used to determine the optimal cut-off values that were determined by maximizing the Youden index (sensitivity + specificity − 1). Results with two-tailed p-values of less than 0.05 were considered significant.

## 3 Result

### 3.1 Baseline characteristics

A total of 305 patients met the entry criteria: 141 IHCs and 164 NA-experienced patients. There were 66 IHCs treated with Peg-IFNα-2b monotherapy (Peg-IFN group) and the other 75 patients without any treatment as controls (untreated group). Among the 164 patients treated with nucleoside analogs, 85 patients received add-on Peg-IFNα-2b (NA add-on Peg-IFN group), and the other 79 patients continued to be treated with nucleoside analogs alone (NA group). **Figure 1** displays the flowchart of participant recruitment in the present study. In the Peg-IFN group, seven patients were lost to follow-up due to poor compliance, two patients stopped the treatment at 12 weeks because of ALT > 10 ULN, and another two patients stopped the Peg-IFN injection at 24 weeks because of agranulocytosis (one patient) and hyperthyroidism (one patient). Also, in the NA add-on Peg-IFN group, seven withdrew because of missing data, and eight patients discontinued treatment due to ALT > 10 ULN (three patients), insomnia (three patients), and hyperthyroidism (two patients). There was no difference in the type of NAs used by the NA add-on Peg-IFN group and NA group, and the baseline characteristics of gender, baseline level of ALT, AST, HBV DNA, HBsAg, white blood cells, AFP, and fibrosis scoring, except age, were generally matched among the four groups (**Table 1**). After further analysis, it was found that the difference of age mainly existed in the Peg-IFN group *vs.* untreated group (p = 0.005) and Peg-IFN group *vs.* NA add-on Peg-IFN group (p = 0.06).

**Table 1:** Baseline of the characteristics of the study population.

Characteristics/treatment	Peg-IFN group (n = 55)	Untreated group (n = 70)	NA add-on Peg-IFN group (n = 70)	NA group (n = 75)	p-Value
Male (%)	32 (58.2%)	47 (67.1%)	53 (75.7%)	52 (69.3%)	0.284
Age, mean ± SD	38.6 ± 11.6	42.5 ± 8.1	41.1 ± 9.2	42.6 ± 6.6	0.013
Nucleoside analogs					0.901
TDF (%)			21 (30.0%)	20 (26.7%)	
ETV (%)			37 (52.9%)	42 (56.0%)	
TAF (%)			12 (17.1%)	13 (17.3%)	
HBsAg at week 0					0.120
<100 IU/mL (%)	37 (67.2%)	45 (64.3%)	37 (52.9%)	40 (53.3%)	
100–250 IU/mL (%)	10 (18.2%)	17 (24.3%)	18 (25.7%)	16 (21.3%)	
250–500 IU/mL (%)	5 (9.1%)	6 (8.6%)	7 (10.0%)	11 (14.7%)	
500–1,000 IU/mL (%)	3 (5.5%)	2 (2.8%)	8 (11.4%)	8 (10.7%)	
HBeAg status at week 0					1.000
Negative (%)	55 (100.0%)	70 (100.0%)	70 (100.0%)	75 (100.0%)	
HBV DNA at week 0					0.253
<100 IU/mL (%)	41 (74.5%)	50 (71.4%)	62 (88.6%)	63 (84.0%)	
100–1,000 IU/mL (%)	9 (16.4%)	14 (20.0%)	4 (5.7%)	7 (9.3%)	
1,000–2,000 IU/mL (%)	5 (9.1%)	6 (8.6%)	4 (5.7%)	5 (6.7%)	
ALT, U/L, median (Q1–Q3)	25.0 (16.0, 35.0)	23.5 (18.8, 31.3)	27.0 (18.8, 33.5)	24.0 (19.0, 31.0)	0.782
AST, U/L, median (Q1–Q3)	23.0 (19.0, 29.0)	23.0 (20.0, 26.0)	24.0 (19.0, 28.3)	24.0 (20.0, 29.0)	0.634
WBC, 10^9^/L, median (Q1–Q3)	5.5 (4.6, 6.6)	6.0(5.0, 7.2)	5.7 (5.0, 6.9)	5.6 (4.8, 7.0)	0.151
TB, μmol/L, median (Q1–Q3)	10.7 (8.3, 14.6)	11.1 (7.9, 14.4)	12.1 (8.3, 15.8)	12.2 (9.6, 16.9)	0.200
A, g/L, median (Q1–Q3)	46.8 (44.9, 48.5)	46.5 (45.0, 48.1)	47.3 (44.1, 48.7)	46.8 (44.9, 49.0)	0.915
N, 10^9^/L, median (Q1–Q3)	2.9 (2.5, 3.9)	3.4 (2.7, 4.3)	3.4 (2.8, 4.0)	3.5 (2.6, 4.2)	0.162
PLT, 10^9^/L, median (Q1–Q3)	215 (184, 245)	195 (169, 228)	197 (168, 229)	198 (174, 234)	0.244
AFP, μg/L, median (Q1–Q3)	3.2 (2.1, 4.3)	3.4 (2.6, 4.3)	3.3 (2.5, 4.0)	2.9 (2.5, 4.1)	0.550
Fibrosis scoring, kPa, median (Q1–Q3)	5.7 (4.5, 6.3)	5.3 (4.6, 6.0)	5.3 (4.6, 6.2)	5.8 (4.8, 6.5)	0.214

SD, standard deviation; TDF, tenofovir disoproxil fumarate; ETV, entecavir; TAF, tenofovir alafenamide; HBeAg, hepatitis B e-antigen; HBsAg, hepatitis B surface antigen; HBV DNA, hepatitis B virus-deoxyribonucleic acid; ALT, alanine aminotransferase; AST, aspartate aminotransferase; WBC, white blood cell; TB, total bilirubin; A, albumin; N, neutrophils; PLT, platelets; AFP, alpha-fetoprotein; Peg-IFN, pegylated interferon; NA, nucleoside analog.

### 3.2 HBsAg clearance

**Figure 2:**
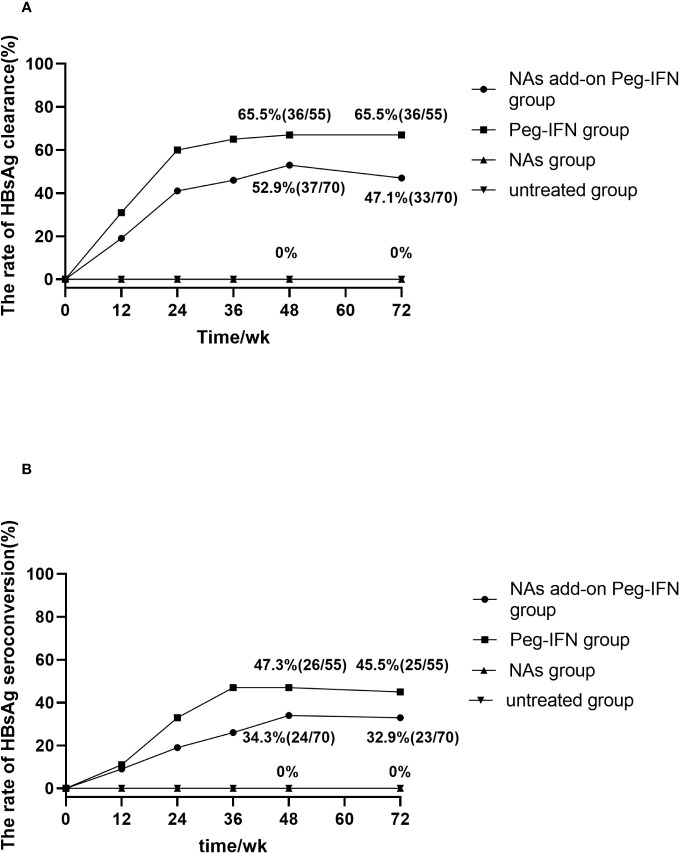
**(A)** Accumulated rates of HBsAg loss. At 72 weeks, HBsAg loss was 65.5% and 47.1% in Peg-IFN group and NA add-on Peg-IFN group, respectively, with the former higher than the latter (p = 0.041). No patient achieved HBsAg loss in the NA group and untreated group, which indicated that pegylated interferon can induce higher HBsAg loss rates (all p< 0.001). **(B)** Accumulated rates of HBsAg seroconversion. At 72 weeks, HBsAg seroconversion was 45.5% in the Peg-IFN group, 32.9% in the NA add-on Peg-IFN group, 0% in the NA group, and 0% in the untreated group. The rates of HBsAg disappearance in the NA group and untreated group were significantly lower than those of two Peg-IFN groups (all p< 0.001), but no significant difference was found between Peg-IFN group and NA add-on Peg-IFN group (p = 0.151). HBsAg, hepatitis B surface antigen; Peg-IFN, pegylated interferon; NA, nucleoside analog.

During the 48-week research period, 36 of 55 patients in the Peg-IFN group achieved HBsAg clearance (65.5%, 36/55). At the same time, the rate of HBsAg clearance was 52.9% (37/70) in the NA add-on Peg-IFN group, with no statistical difference between the two groups (p = 0.156). In Peg-IFN-free groups (NA group and untreated group), there were no patients who achieved HBsAg loss (**Figure 2A**).

At the follow-up of 72 weeks, 36 patients in the Peg-IFN group achieved HBsAg serological clearance (65.5%, 36/55), and 33 patients in the NA add-on Peg-IFN group achieved HBsAg clearance (47.1%, 33/70), with the former significantly higher than the latter (p = 0.041). Meanwhile, no patients achieved HBsAg disappearance at 72 weeks in the NA group and untreated group (**Figure 2A**). Among those patients who did not achieve HBsAg loss, there were five patients (two patients in the Peg-IFN group and three patients in the NA add-on Peg-IFN group) who obtained HBsAg clearance at 72 weeks. At the same time, two patients in the Peg-IFN group experienced HBsAg seroconversion at 48 weeks with a level of HBsAb<100/mL but developed HBsAg relapse at 72 weeks. Seven patients in the NA add-on Peg-IFN group had HBsAg relapse, four of them achieved HBsAg seroconversion, but the level of HBsAb was less than 100 IU/mL at 48 weeks.

### 3.3 HBsAg seroconversion

Twenty-six (47.3%, 26/55) patients in the Peg-IFN group and 24 patients (34.3%, 24/70) in the NA add-on Peg-IFN group achieved HBsAg seroconversion at 48 weeks, but there was no significant difference between the two groups (p = 0.141) (**Figure 2B**).

At 72 weeks, the HBsAg seroconversion rates in the Peg-IFN group and NA add-on Peg-IFN group were 45.5% (25/55) and 32.9% (23/70), respectively (p = 0.151) (**Figure 2B**). A total of six patients who had HBsAg seroconversion but with a level of HBsAb less than 100 IU/mL had HBsAg relapse, with two cases in the Peg-IFN group and four cases in the NA add-on Peg-IFN group. At the same time, four patients (one in the Peg-IFN group and three in the NA add-on Peg-IFN group) with HBsAg loss developed HBsAg seroconversion. Among the untreated and NA monotherapy patients, none achieved HBsAg seroconversion (**Figure 2B**). These results indicated that Peg-IFNα-2b could significantly improve the rate of HBsAg clearance and HBsAg seroconversion compared with NA monotherapy or natural state (all p< 0.001).

### 3.4 Decline of HBsAg in predicting clinical cure

**Figure 3:**
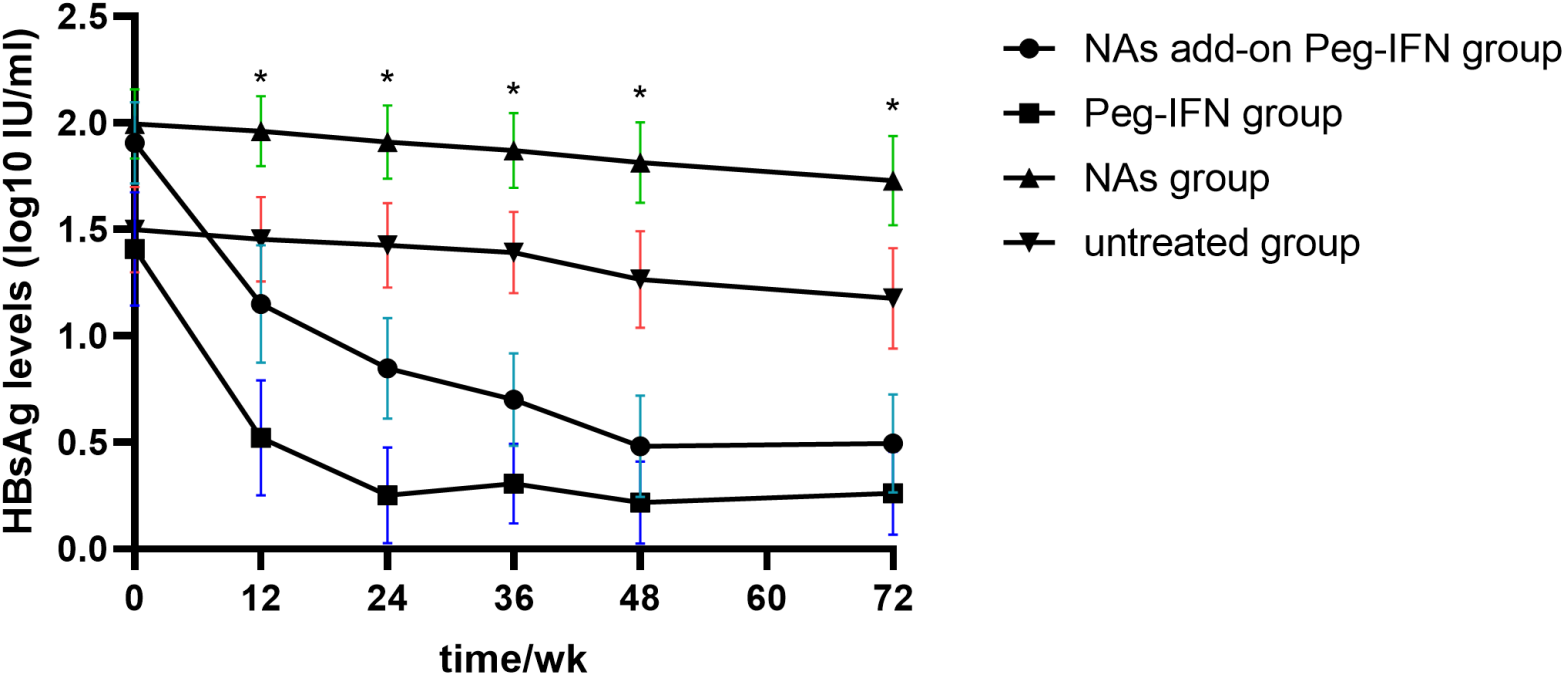
Kinetic reduction in hepatitis B surface antigen (HBsAg) level. Mean serum HBsAg level was reduced to significantly lower levels in the NA add-on Peg-IFN group and Peg-IFN group compared with the NA group and untreated group at weeks 12, 24, 36, 48, and 72. * p< 0.05. NA, nucleoside analog.

The results in **Figure 3** revealed that HBsAg significantly decreased compared with baseline levels in all patients who received Peg-IFNα-2b, especially at 12 and 24 weeks. However, the level of HBsAg in those patients who did not receive Peg-IFNα-2b showed no significant changes during the entire research period. We found that the decline of HBsAg from baseline to 12 weeks and 24 weeks of more than 80% [OR = 0.993, IC: 0.989–0.997, p = 0.001] and 98% [OR = 50.815, IC: 6.154–419.603, p = 0.006] can predict HBsAg clearance (**Table 2**). The HBsAg clearance rate can reach 88.6% (62/70) in those HBsAg levels that decline over 80% at 12 weeks.

**Table 2:** (A) The predictive factors of HBsAg clearance at week 48 in all Peg-IFN-treated patients and (B) prediction of HBsAg loss at week 48 in patients who received Peg-IFN treatment.

(A) Predictors	Univariate analysis			Multivariate analysis			
	OR	95%CI	p	OR	95%CI		p
Age, years	0.984	0.950–1.019	0.369				
Gender (male)	2.253	0.999–5.082	0.005	1.214	0.350–4.211		0.760
Treatment (NA add-on Peg-IFN)	1.634	0.784–3.406	0.190	0.382	0.108–1.355		0.136
Baseline HBsAg level IU/mL	0.998	0.991–0.997	0.000	0.993	0.989–0.997		0.001
HBsAg level at 12 weeks, IU/mL	0.985	0.978–0.992	0.000	1.002	0.992–1.011		0.722
HBsAg level at 24 weeks, IU/mL	0.965	0.947–0.984	0.000	1.006	0.987–1.026		0.527
Baseline HBV DNA<100 IU/mL	1.355	0.504–3.640	0.547				
HBV DNA<100 IU/mL at week 12	0.313	0.280–3.552	0.349				
HBsAg decline from baseline to week 12, %	17.192	5.945–49.717	0.000	4.221	1.156–15.409		0.029
HBsAg decline from baseline to week 24, %	131.816	48.054–361.586	0.000	50.815	6.154–419.603		0.006
Baseline ALT, *ULN	1.013	0.988–1.040	0.314				
ALT elevation at week 12, *ULN	1.076	0.917–1.263	0.371				
ALT elevation at week 12, *ULN	1.165	0.868–1.563	0.310				
(B) Prediction model	Area	Cut-off point	Sensitivity		Specificity	p	
Baseline HBsAg level IU/mL	0.770	72	77.5%		69.7%	<0.001	
HBsAg decline from baseline to week 12, %	0.877	80	80.3%		83.7%	<0.001	
HBsAg decline from baseline to week 24, %	0.915	98	90.8%		95.9%	<0.001	

OR, odds ratios; CI, confidence interval; HBsAg, hepatitis B surface antigen; HBV DNA, hepatitis B virus-deoxyribonucleic acid; ALT, alanine aminotransferase; ULN, upper limit of normal value; Peg-IFN, pegylated interferon; NA, nucleoside analog.

Therefore, our results showed that a decrease of HBsAg of more than 80% and 98% from baseline to 12 and 24 weeks is an independent predictor of clinical cure.

### 3.5 Baseline HBsAg level in predicting clinical cure

**Figure 4:**
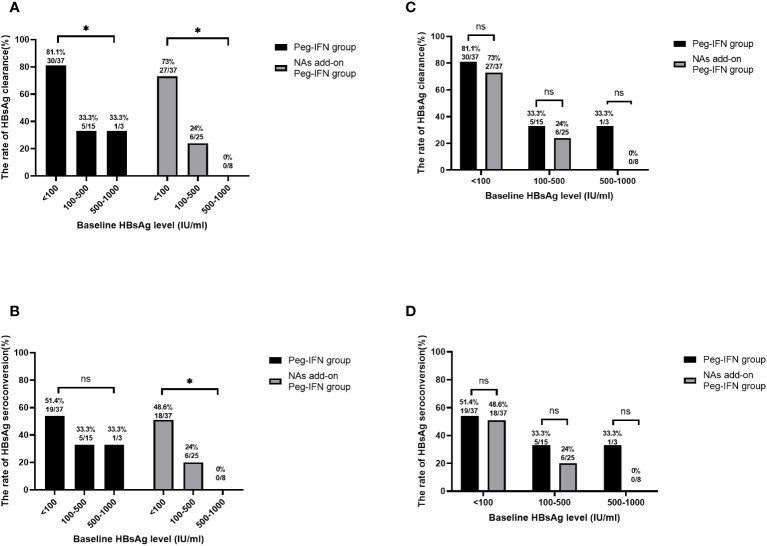
HBsAg clearance based on baseline HBsAg stratification. **(A)** Lower baseline HBsAg levels mean higher HBsAg clearance potential in both the Peg-IFN group and NA add-on Peg-IFN group (both p< 0.05). **(B)** In the Peg-IFN group, there was no significant statistical difference in HBsAg clearance rates with different baseline HBsAg levels (p = 0. 459). In the NA add-on Peg-IFN group, the patients with HBsAg< 100 IU/mL had higher HBsAg seroconversion rates (p< 0.05). **(C, D)** The Peg-IFN group and the NA add-on Peg-IFN group had similar HBsAg clearance rates and HBsAg seroconversion rates based on the same baseline HBsAg stratification (all p > 0.05). * p< 0.05. ns, not significant; HBsAg, hepatitis B surface antigen; Peg-IFN, pegylated interferon; NA, nucleoside analog.

We stratified the patients based on their baseline HBsAg levels to further analyze the impact of baseline HBsAg levels in clinical cure, with the results suggesting that the rates of HBsAg clearance in participants with baseline HBsAg< 100 IU/mL, 100–500 IU/mL, and 500–1,000 IU/mL were 81.1% (30/37), 33.3% (5/15), and 33.3% (1/3), respectively, in the Peg-IFN group (**Figure 4A**). Meanwhile, 19 patients (51.4%, 19/37) in the HBsAg< 100 IU/mL group achieved HBsAg seroconversion. When the baseline HBsAg was at 100–500 IU/mL and 500–1,000 IU/mL, the HBsAg seroconversion rates were 33.3% (5/15) and 33.3% (1/3), respectively (**Figure 4B**). At the same time, the patients in the NA add-on Peg-IFN group with baseline HBsAg below 100 IU/mL had a higher possibility of achieving HBsAg loss (73.0%, 27/37) than those with baseline HBsAg levels of 100–500 IU/mL (24.0%, 6/25) and 500–1,000 IU/mL (0.0%, 0/8) (**Figure 4A**). Consistent with the change in the trend of HBsAg clearance rates, the HBsAg seroconversion rate was also higher in participants with baseline HBsAg levels<100 IU/mL (48.6%, 18/37) than those with baseline HBsAg levels of 100–500 IU/mL (24.0%, 6/25) and 500–1,000 IU/mL (0.0%, 0/8) (**Figure 4B**). The above results reveal that patients with baseline HBsAg below 100 IU/mL have a higher clinical cure rate.

Based on the same baseline HBsAg stratification, there was no significant statistical difference in the HBsAg clearance rate and seroconversion rate between the Peg-IFN group and the NA add-on Peg-IFN group (**Figures 4C, D**).

Of the patients who had baseline HBsAg< 100 IU/mL, 77.0% (57/74) obtained clinical cure in the Peg-IFN group and NA add-on Peg-IFN group. Further analyzing those patients, we found that there were 26 patients (70.3%, 26/37) in the Peg-IFN group and 25 (67.6%, 25/37) in the NA add-on Peg-IFN group who achieved clinical cure within 24 weeks of therapy, but there was no significant statistical difference between the two groups (p = 0.802). Moreover, no patient in the Peg-IFN group experienced HBsAg recurrence after a 24-week follow-up, and the persistence of HBsAg clearance could reach 100%. In the NA add-on Peg-IFN group, three patients developed HBsAg relapse during the follow-up period.

Univariate logistic regression analysis and multivariate logistic regression analysis clarified that HBsAg level was a predictor of HBsAg clearance, and the optimal cut-off point was 72 IU/mL (area under the receiver operating characteristic curve (AUROC) of 0.770, sensitivity of 77.5%, and specificity of 69.7%), which is consistent with the results of the HBsAg stratification (**Table 2**). Consequently, baseline HBsAg<100 IU/mL was considered to be an independent predictor of clinical cure.

### 3.6 Safety

The rates of adverse events were similar in the Peg-IFN group and NA add-on Peg-IFN therapy groups (p > 0.050). The most common adverse events include headache, myalgia, pyrexia, fatigue, alopecia, gastrointestinal symptoms, neutropenia, thrombocytopenia, and ALT elevation (**Table 3**). No serious adverse events happened among the Peg-IFN-treated groups. After the Peg-IFNα-2b was stopped, in the patients who experienced elevated ALT levels during Peg-IFNα-2b therapy, ALT levels gradually returned to normal at the follow-up of 24 weeks.

**Table 3:** Adverse effect of Peg-IFN and NAs.

	Peg-IFN group (n = 55)	NA add-on Peg-IFN group (n = 70)	NA group (n = 75)
Body as whole			
Pyrexia	19 (34.5%)	27 (38.6%)	0 (0.0%)
Headache	50 (91.0%)	58 (82.9%)	0 (0.0%)
Fatigue	21 (38.2%)	36 (51.4%)	5 (5.7%)
Myalgia	43 (78.1%)	60 (85.7%)	0 (0.0%)
Dermatological			
Rash	5 (7.3%)	8 (11.4%)	0 (0.0%)
Pruritus	1 (1.8%)	3 (4.3%)	1(1.4%)
Alopecia	19 (34.5%)	21 (30.0%)	0 (0.0%)
Digestive tract			
Decreased appetite	49 (89.1%)	62 (88.6%)	3 (4.3%)
Diarrhea	4 (7.3%)	5 (7.1%)	0 (0.0%)
Nausea	5 (9.1%)	7 (10.0%)	0 (0.0%)
Dyspepsia	14 (25.5%)	14 (20.0%)	2 (2.9%)
Hematological			
Anemia	18 (32.7%)	28 (40.0%)	2 (2.9%)
Neutropenia	47 (85.5%)	65 (92.9%)	0 (0.0%)
Thrombocytopenia	23 (41.8%)	36 (51.4%)	4 (5.7%)
Psychiatric			
Depression	1 (1.8%)	3 (4.3%)	0 (0.0%)
Anxiety	10 (20.0%)	15 (21.4%)	0 (0.0%)
Insomnia	26 (47.3%)	37 (52.9%)	3(4.3%)

Peg-IFN, pegylated interferon; NAs, nucleoside analogs.

## 4 Discussion

Nowadays, after long-term antiviral therapy, chronic hepatitis B patients can reach a virological suppression status, which can lead to histological improvement and regression of liver fibrosis and cirrhosis (Chang et al., 2010; Marcellin et al., 2013). However, with the emergence of new therapeutic options, the clinical cure of hepatitis B has become possible and can greatly reduce hepatitis B-related complications. A large retrospective cohort study revealed that patients who achieved HBsAg clearance on top of complete viral suppression may have a lower risk of HCC (Yip et al., 2019).

Achieving a clinical cure is still difficult for CHB patients. A large number of studies have confirmed that NA-experienced patients can achieve HBsAg clearance through 48 weeks of Peg-IFN therapy. Hu et al. (Hu et al., 2021) demonstrated that 11.6% of patients can obtain HBsAg loss after a 48-week course of Peg-IFN in combination with tenofovir disoproxil fumarate (TDF). Another study (Boglione et al., 2016; Wu et al., 2020) showed that the rates of HBsAg clearance can rise to 37.4% in those patients with HBsAg< 1,500 IU/mL. Some studies (Cao et al., 2017; Wu et al., 2021) also found that the rate of HBsAg clearance can reach 29.8% to 47.1% in the IHC population. Recently, Zhang et al. (Zhang et al., 2023) conducted a study about the comparison of Peg-IFN in NA-experienced patients and IHCs. They showed that the rates of HBsAg loss were 36.8% and 32.6% in the IHC group and NA add-on Peg-IFN group, respectively, after 24-week treatment, but their research lacked Peg-IFN-free control groups. Therefore, our study not only compared the clinical cure of Peg-IFNα-2b between NA-experienced patients and IHCs but also set the Peg-IFN-free control groups to reflect the efficacy of Peg-IFNα-2b in reducing HBsAg level.

Our research showed that the rates of HBsAg loss at 48 weeks were 52.9% and 65.5% in the NA add-on Peg-IFN group and Peg-IFN group, respectively, and the rates changed to 47.1% and 65.5%, respectively, at 72 weeks. Additionally, IHCs showed a significantly better treatment response than NA-experienced patients at 72 weeks. On the one hand, this may be related to patients in the Peg-IFN group being younger. Lee et al. (Lee et al., 2018) believe that age< 35 years is a predictive factor of HBsAg clearance for Peg-IFN-treated patients. After achieving sustained off-treatment virological response, HBsAg loss rates were higher than 30% in 5 years among patients with younger age, which suggested that the younger patients experienced better efficacy of Peg-IFN therapy. On the other hand, there were more patients who had HBsAg relapse in the NA add-on Peg-IFN group at 72 weeks. We suspect that the reason for the higher relapse rate in the NA add-on Peg-IFN group may be related to the discontinuation of NAs after HBsAg clearance. In view of the higher HBsAg relapse rate in the NA add-on Peg-IFN group, we believe that HBsAg relapse may be reduced by prolonging Peg-IFN treatment for NA-experienced patients. During the entire study period, no patient in the NA group or untreated group experienced HBsAg clearance or seroconversion, which is similar to the results of previous related studies, suggesting that Peg-IFN-based treatment can significantly improve HBsAg clearance (Chi et al., 2014; de Niet et al., 2017). We believe that both IHCs and NA-experienced patients can achieve high HBsAg clearance rates and seroconversion rates through Peg-IFN treatment, so Peg-IFN may also be beneficial for IHCs to achieve clinical cure.

Emerging data suggest that baseline characteristics and the extent of early on-treatment HBsAg reduction may predict the possibility of HBsAg loss with Peg-IFN treatment (Moucari et al., 2009; Rijckborst et al., 2010; Yeo et al., 2019). We found that baseline HBsAg level and HBsAg decline rates from baseline to 12 weeks and 24 weeks were all useful independent predictors of HBsAg clearance, which have been supported by many previous studies in individualized on-treatment decision-making (Hu et al., 2018; Zheng et al., 2019; Zhang et al., 2023). In our study, the decline of HBsAg from baseline to 12 weeks and 24 weeks of more than 80% and 98% is a strong predictor of HBsAg clearance. The HBsAg clearance rate was negatively correlated with HBsAg levels in the Peg-IFN group and NA add-on Peg-IFN group, which was similar to the results of Li et al. (Li et al., 2016) and Wu et al. (Chang et al., 2010; Marcellin et al., 2013). A low HBsAg level before starting Peg-IFN treatment is the most important prognostic factor for achieving HBsAg loss (Ning et al., 2014). HBsAg level< 100 IU/mL has been cited by many studies as a significant milestone in HBsAg reduction because it is more frequently associated with HBsAg loss (Jeng et al., 2018). A cohort research (Li et al., 2016) has confirmed that patients with HBsAg< 100 IU/mL have a high clinical cure rate. Consistent with the former study, our study found that 77% (Peg-IFN group, 81.1%; NA add-on Peg-IFN group, 73%) of the patients with the baseline HBsAg<100 IU/mL obtained HBsAg clearance at 48 weeks, and the optimal cut-off value was baseline HBsAg below 72 IU/mL. Therefore, the patients with baseline HBsAg< 100 IU/mL and negative HBeAg can be clinically cured by Peg-IFN therapy. The current standard Peg-IFN treatment course is 48 weeks. A study (Zeng et al., 2020) that enrolled IHCs with HBsAg< 20 IU/mL showed that 93.8% of participants achieved HBsAg loss within 24 weeks of Peg-IFN therapy. Similarly, our research revealed that more than 70% patients with HBsAg< 100 IU/mL (Peg-IFN group, 70.3%; NA add-on Peg-IFN, 73.0%) had obtained HBsAg clearance within 24-week Peg-IFN therapy, and few of them had HBsAg relapse during 24 weeks of follow-up, which suggested that low baseline HBsAg levels might be associated with short therapeutic time. Therefore, whether the patients with baseline below 100 IU/mL need to shorten the therapy time of Peg-IFN still require more studies to be confirmed.

This study also has some limitations. First, this is a non-randomized cohort study, so the patients were not evenly distributed in each group. The patients treated with Peg-IFN had a strong desire for HBsAg clearance, but the patients in the NA group and the untreated group, due to economic reasons or slow progression of the disease, refused to use Peg-IFN (Liu et al., 2016). Second, the patient population was relatively small, and all patients were recruited from a single center. Therefore, more large-sample, multi-center studies are needed in the future to further illustrate the above findings. Third, long-term follow-up is required to evaluate the durability of HBsAg clearance and the incidence of liver cirrhosis and HCC after stopping treatment. Recently, some studies revealed that HCC incidence can be reduced to 1% after nucleoside-induced or spontaneous HBsAg clearance (Yip et al., 2019; Lok et al., 2020). Therefore, it is necessary to follow up these patients to further evaluate the efficacy and safety of Peg-IFN.

In conclusion, our study suggested that Peg-IFN treatment can significantly improve the HBsAg clearance rate of IHCs and NA-experienced patients with low HBsAg levels (baseline HBsAg< 1,000 IU/mL), especially those patients whose HBsAg is below 100 IU/mL.

## Data availability statement

The raw data supporting the conclusions of this article will be made available by the authors, without undue reservation.

## Ethics statement

The studies involving humans were approved by Ethics Committee of the Second Affiliated Hospital of Chongqing Medical University. The studies were conducted in accordance with the local legislation and institutional requirements. The participants provided their written informed consent to participate in this study.

## Author contributions

CW: Data curation, Formal analysis, Investigation, Methodology, Writing – original draft, Writing – review & editing. YW: Data curation, Investigation, Writing – original draft. HT: Data curation, Investigation, Writing – original draft. YL: Investigation, Methodology, Writing – review & editing. ZW: Investigation, Writing – review & editing, Resources. DC: Investigation, Resources, Writing – review & editing. ZZ: Investigation, Resources, Writing – review & editing. XS: Resources, Writing – review & editing, Conceptualization, Project administration, Supervision.
